# Compartment Syndrome Following Extravasation of Contrast: A Case Report

**DOI:** 10.7759/cureus.99426

**Published:** 2025-12-16

**Authors:** Bernardo R Cavadas, Miguel Veríssimo, Inês O Pires, Luís Vieira, Luís M Ribeiro

**Affiliations:** 1 Plastic and Reconstructive Surgery, Unidade Local de Saúde de São José, Lisbon, PRT; 2 Plastic and Reconstructive Surgery, Unidade Local de Saude de São José, Lisbon, PRT

**Keywords:** acute compartment syndrome, contrast media complications, fasciotomy, hand compartment syndrome, iatrogenic injury, intravenous contrast extravasation, surgical decompression

## Abstract

Acute compartment syndrome is a rare but serious complication of intravenous contrast extravasation, which can cause tissue ischemia and necrosis if not recognized and treated promptly.

We report the case of a 56-year-old female with a history of breast cancer who developed acute compartment syndrome of the right hand and forearm after extravasation of approximately 20-30 mL of iodinated contrast agent administered via the dorsal hand during a CT scan. The patient presented with progressive swelling, pain disproportionate to injury, impaired digital perfusion, and limited mobility. Based on clinical findings, urgent surgical decompression via fasciotomy of the hand, wrist, and forearm compartments was performed with successful restoration of perfusion and function. Postoperative recovery was uneventful, although mild carpal tunnel syndrome developed on follow-up.

This case highlights the importance of early recognition of intravenous contrast extravasation as a potential cause of acute compartment syndrome, especially in high-risk patients such as those with fragile veins, prior chemotherapy or obesity. Prompt diagnosis and immediate fasciotomy are crucial to prevent permanent functional impairment and severe morbidity. Awareness and vigilance among healthcare professionals administering contrast agents can improve patient outcomes through timely intervention.

## Introduction

Compartment syndrome is an infrequent but potentially catastrophic condition characterized by an elevation in pressure within a closed compartment, resulting in neuromuscular damage, skin loss, and, in severe cases, amputation [1,2].

Traumatic injuries and burns are among the most common causes of compartment syndrome [3,4]. However, iatrogenic causes, including the use of tourniquets, pressurised infusion pumps, anticoagulation therapy, and extravasation of intravenous drugs or fluids (particularly in limbs with compromised lymphatic or venous drainage), are becoming more frequent [5]. Extravasation is the accidental injection or leakage of fluid and drugs into the extravascular or subcutaneous space [6].

Early diagnosis and prompt treatment are crucial for managing acute compartment syndrome and minimizing functional loss. In conscious and cooperative patients, diagnosis can be made clinically by obtaining a comprehensive medical history and conducting a physical examination [7]. The classic 5 Ps-pain disproportionate to the injury, pallor, paresthesia, paralysis, and pulselessness-are commonly described clinical signs in compartment syndrome. Among these, disproportionate pain is the earliest and most reliable indicator, while the others typically manifest later and may indicate advanced ischemic damage [8-10]. Immediate decompressive fasciotomy is mandatory once the diagnosis is confirmed [11].

In the present report, we describe a rare case of compartment syndrome of the hand and forearm following extravasation of intravenous contrast.

## Case presentation

We report the case of a 56-year-old Caucasian female patient with a history of breast cancer who had undergone mastectomy, adjuvant chemotherapy, and hormonal therapy. Her past medical history also included obesity and arterial hypertension. There was no relevant family history or known allergies.

Prior to the procedure, the right upper limb was neurovascularly intact and fully functional, despite the patient's history of axillary lymph node dissection. As part of her oncologic follow-up, she underwent a thoracoabdominal and pelvic CT scan with intravenous contrast at an external facility. The contrast was administered via a catheter in the dorsal aspect of the right hand. During the injection, contrast extravasation occurred, resulting in the immediate onset of pain and progressive edema with significant functional limitation. Consequently, she was urgently referred to our Plastic Surgery Emergency Department, and surgical decompression was performed approximately six hours after the extravasation event.

The CT scan was performed following standard protocols. However, it is noteworthy that the patient had difficult peripheral venous access. The contrast agent used was non-ionic Iopromide (Ultravist® 370, injectable solution). An automatic injector was utilized, and administration was halted promptly upon suspicion of extravasation. The estimated injected volume was approximately 20 to 30 mL.

**Figure 1:**
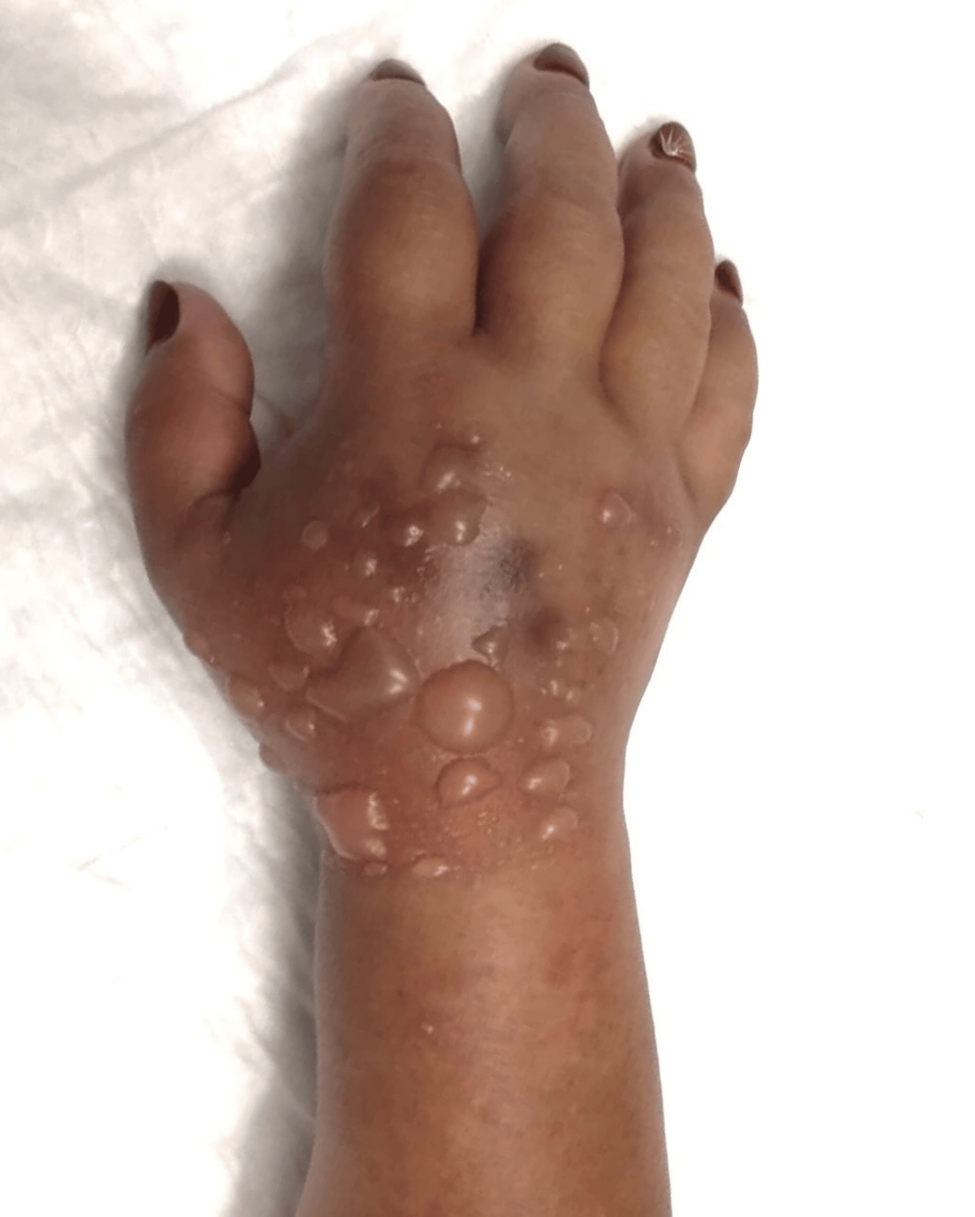
Dorsal view of the right hand, wrist and forearm before surgery. The image shows an exuberant edema and blisters in the dorsum of the hand with impending ischemia of the fingers.

**Figure 2:**
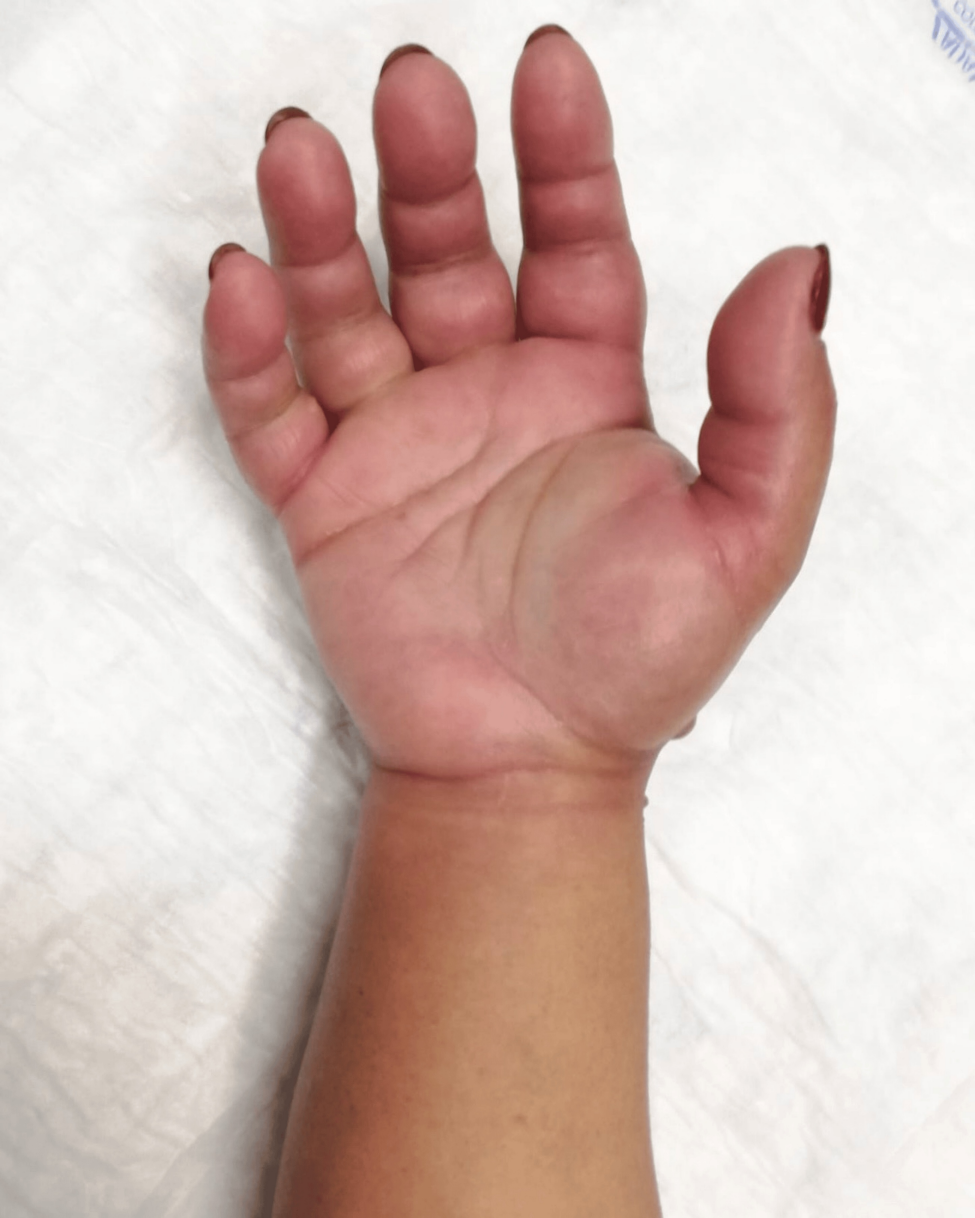
Volar view of the right hand, wrist, and forearm before surgery. The image demonstrates diffuse edema and skin tension.

Upon admission to the emergency department, the patient presented with significant edema and blistering on the dorsum of the right hand (Figure 1), extending to the volar aspect of the wrist (Figure 2). This was associated with poor digit perfusion, , delayed capillary refill time (>4 seconds), and limited mobility due to severe pain (rated 9/10 on the Visual Analog Scale). There were no alterations on the laboratory and imaging examinations. Invasive compartment pressure assessment was not performed as this was not available in this Hospital.

**Figure 3:**
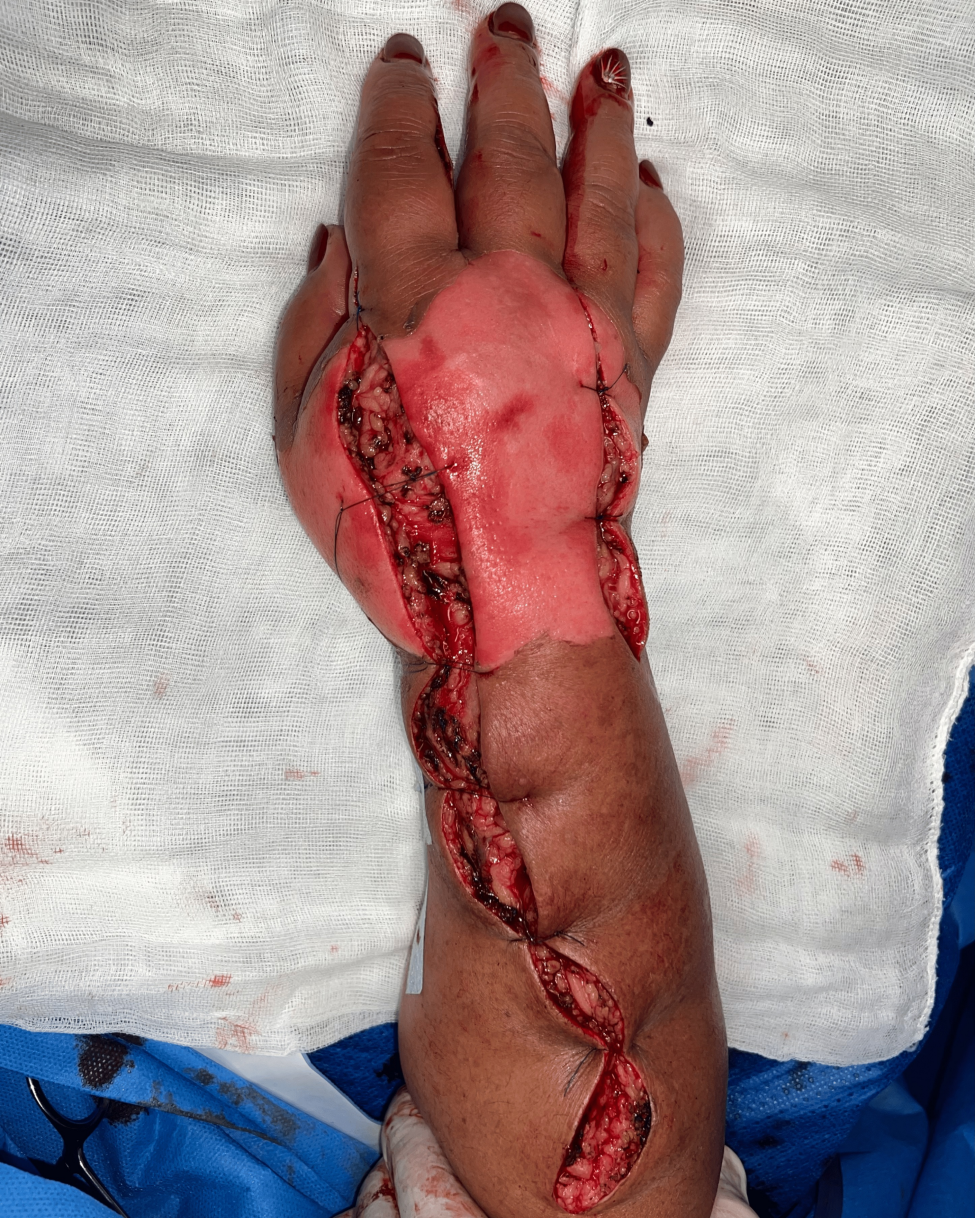
Postoperative dorsal view immediately following decompressive fasciotomies. The image showing the release of the dorsal and interosseous compartments.

**Figure 4:**
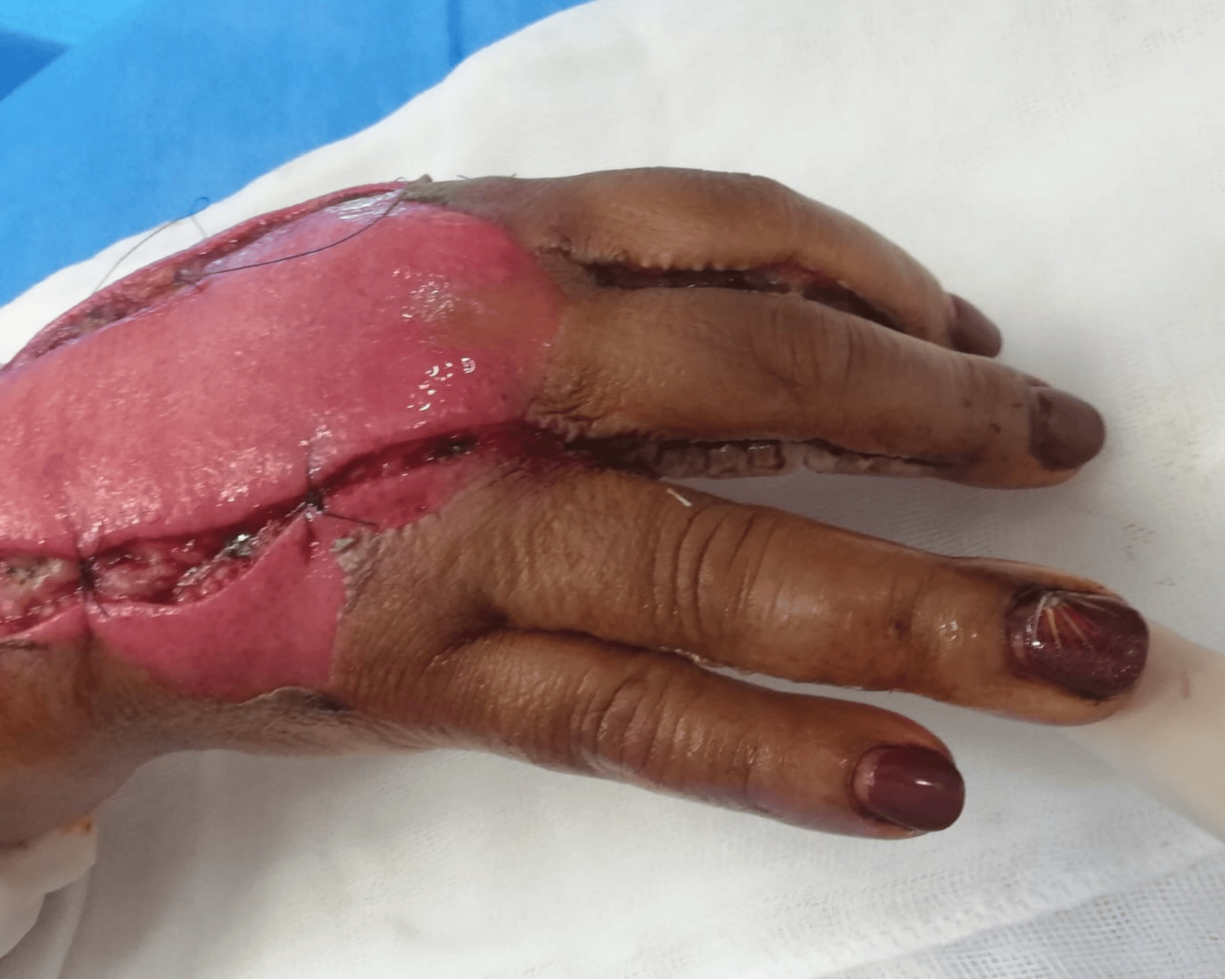
Intraoperative view of the right hand demonstrating dorsal hand and finger fasciotomies. The image also show a release of the interosseous compartments and lateral digital incisions, allowing decompression of the dorsal hand and digits.

Based on clinical evaluation, acute compartment syndrome was diagnosed, and the patient was immediately transferred to the operating room. The patient underwent decompression incisions on the dorsum of the hand, wrist, and forearm with release of the interosseous compartments as well as the dorsal compartment of the forearm (Figure 3). Additionally, lateral digital incisions were made on the ulnar border of the 2nd, 3rd and 4th fingers (Figure 4). At the end of the procedure, the skin of the dorsum of the hand presented doubtful viability but the fingers were well perfused, and the compartments were soft. 

Postoperatively, the incisions were dressed, and the patient was monitored in the inpatient ward with limb elevation and intravenous antibiotics. Early range of motion exercises were initiated. By the third postoperative day, edema had resolved, and the dorsal hand wound presented with healthy granulation tissue. The patient then underwent a second surgical procedure for delayed primary closure of all fasciotomy incisions (fingers, hand, and forearm) without tension. She was discharged five days after admission.

**Figure 5:**
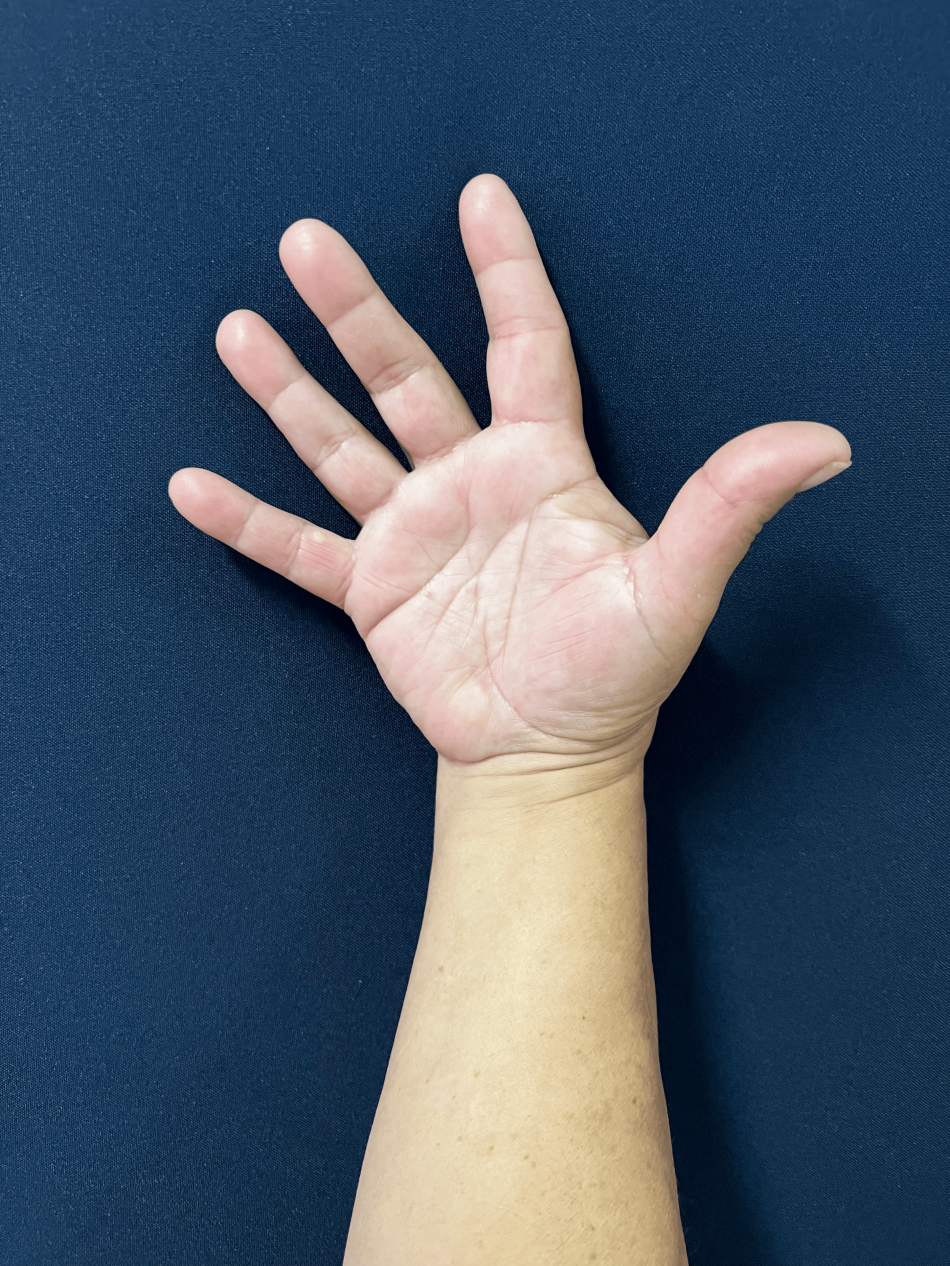
Clinical photograph at six months follow-up. The figure demonstrating full active extension and abduction of the right hand and fingers, with preserved range of motion and satisfactory functional recovery.

**Figure 6:**
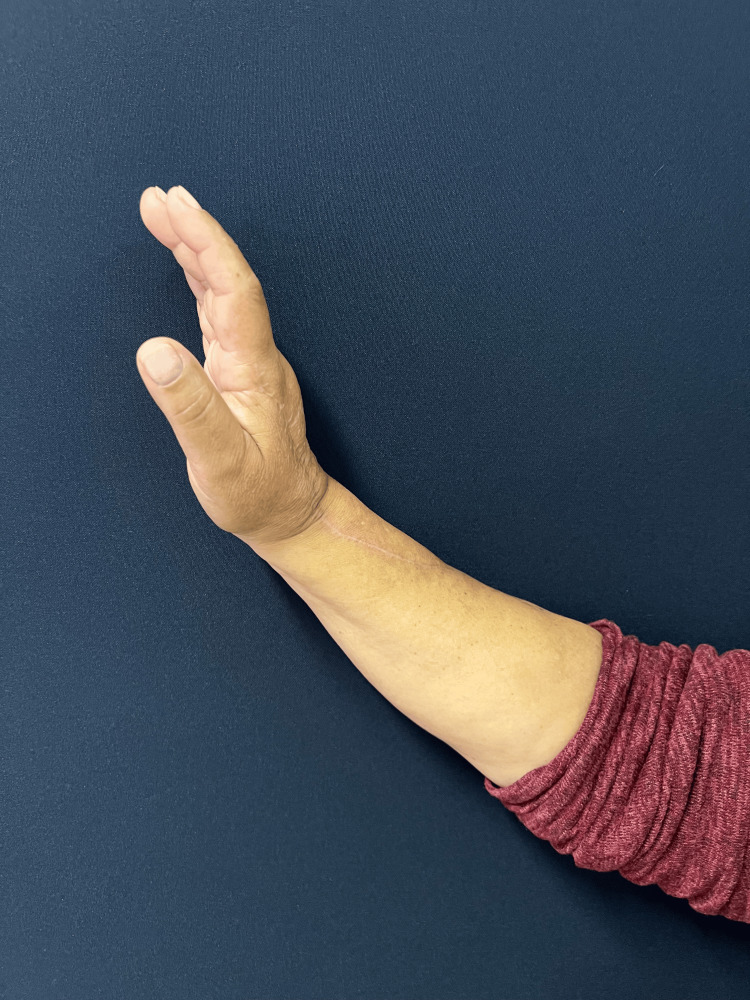
Lateral view of the right hand and forearm at six-month follow-up. The image demonstrates good wrist extension and wa ell-healed surgical scar.

**Figure 7:**
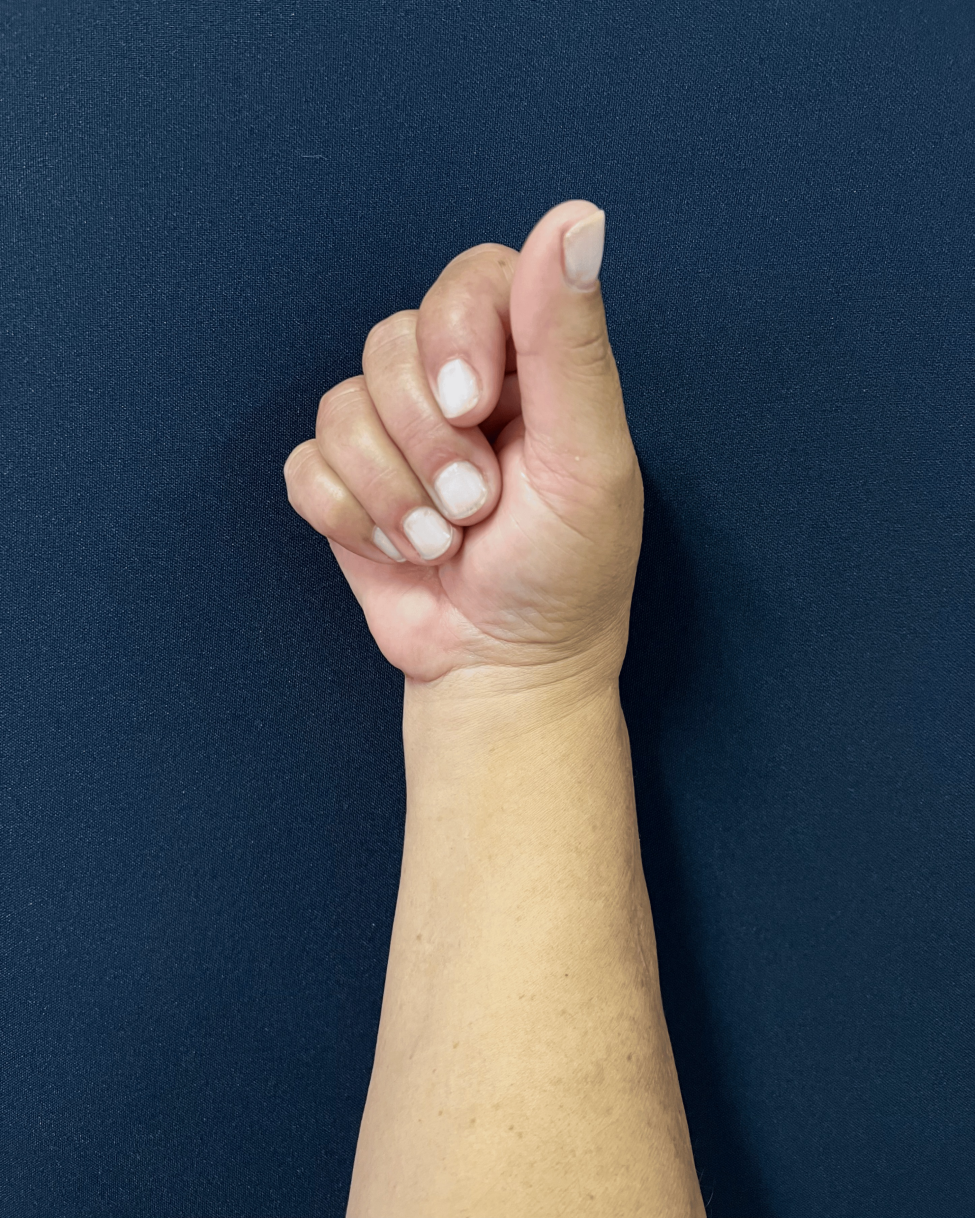
Clinical photograph at six-month follow-up. The image demonstrating near-complete active flexion of the right fingers and thumb, with only mild limitation at end-range, consistent with good overall motor recovery.

**Figure 8:**
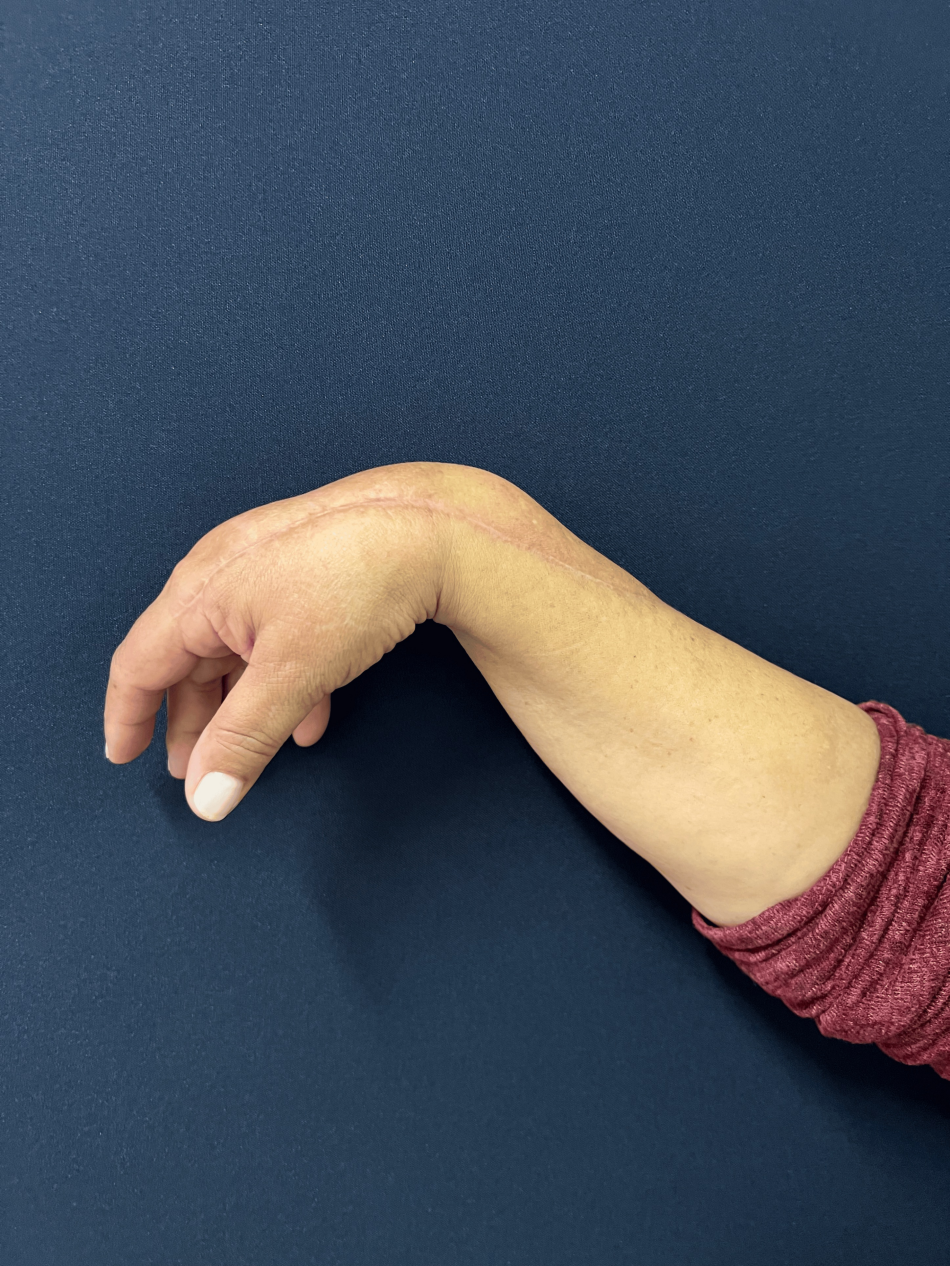
Lateral view of the right hand and forearm at six-month follow-up. The figure demonstrating good wrist flexion, with the healed scar not limiting range of motion.

**Figure 9:**
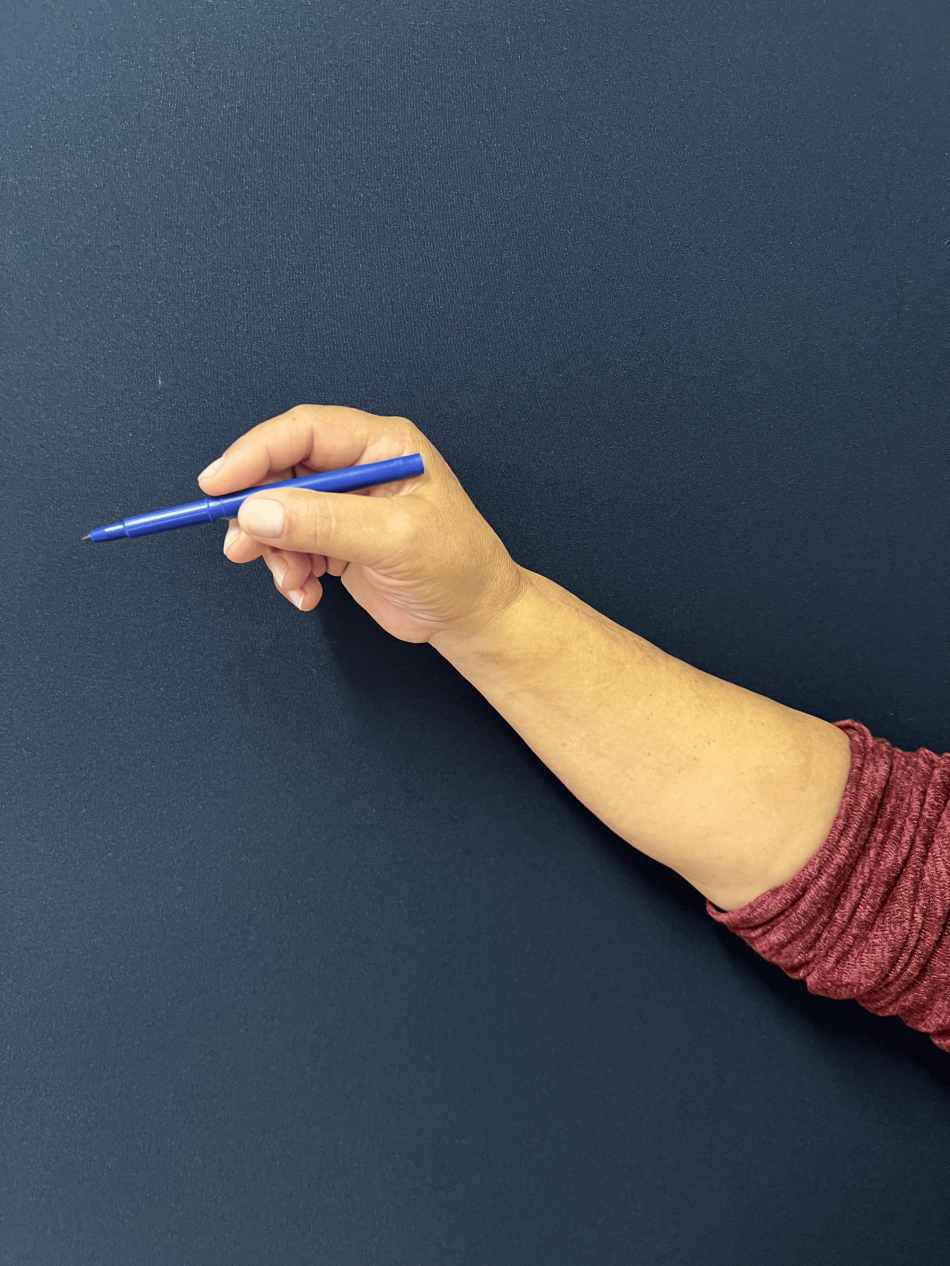
Clinical photograph demonstrating a pinch grip test. The image shows with the patient holding a pen to illustrate preservation of fine motor function and precision pinch.

The follow-up was unremarkable, with satisfactory sensory and motor recovery at 6 months postoperatively. The patient regained full active extension and abduction (Figure 5) and near-complete flexion of the fingers (Figure 6). Wrist mobility was preserved, showing good extension (Figure 7) and flexion (Figure 8). Furthermore, fine motor function was intact, as evidenced by a preserved pinch grip (Figure 9). This complication did not affect the oncological treatment, nor did it result in any delays in chemotherapy or hormone therapy treatments.

At 12 months postoperatively, clinical examination revealed signs consistent with carpal tunnel syndrome, including mild global motor weakness of the right hand and hypoesthesia in the index and middle fingers. Electromyography confirmed the diagnosis, necessitating surgical decompression. At the 15-month follow-up, the patient completed the Disabilities of the Arm, Shoulder, and Hand (DASH) questionnaire, scoring 44.2 [12]. This score indicates a moderate level of disability, confirming that, despite successful limb salvage, functional sequelae persisted. However, the patient noted that these limitations did not significantly affect her daily activities.

## Discussion

Although rare, iatrogenic events such as extravasation of drugs or contrast solutions can lead to a range of clinical presentations, from mild local reactions to acute compartment syndrome and soft tissue necrosis [13]. The incidence of contrast extravasation is reported to range from 0.24% to 1.2% [14,15]. Therefore, a high index of suspicion should be maintained when encountering such cases, particularly in patients who are obese, have small or fragile veins, or have disseminated skin diseases such as psoriasis. Other risk factors include catheter placement in areas with little soft tissue, such as the dorsum of the hand, or in limbs with peripheral vascular disease or compromised lymphatic drainage [16]. In the presented case, multiple risk factors were present: obesity, fragile veins due to prior chemotherapy, compromised lymphatic drainage (sequela of axillary dissection), and catheter placement on the dorsum of the hand.

Extravasation of contrast typically causes mild discomfort, erythema, and swelling. However, if a large volume of fluid, particularly a hyperosmolar contrast solution, is injected at high speed, it can result in extensive tissue damage and acute compartment syndrome [16].

During the physical examination, the patient presented with typical symptoms of compartment syndrome, including pain disproportionate to the injury, pain on passive finger movement, tense compartments upon palpation, and decreased digital perfusion. In such scenarios, adjunctive diagnostic tests are often unnecessary, and prompt, effective decompression of the affected compartments must be the priority. In this case, the surgical team performed dorsal hand incisions aligned with the 2nd and 4th intermetacarpal spaces to decompress the interosseous compartments. Additional incisions were made on the ulnar border of the index, middle, and ring fingers to ensure proper digital perfusion. Furthermore, a dorsal fasciotomy was performed on the forearm to release the dorsal compartment.

Healthcare professionals, including radiologists, nurses, and imaging technicians, should be aware of the potential symptoms of contrast extravasation and the importance of routinely verifying catheter function prior to infusion, which can be achieved by performing a test bolus injection [14]. It is crucial to recognize symptoms promptly to ensure fasciotomies are performed before permanent damage occurs. If symptoms arise in a clinic or hospital lacking the necessary surgical capabilities, expedited transport to an appropriate facility is mandatory, as delayed treatment can lead to catastrophic outcomes. Ultimately, decompression should be performed as soon as possible, as acute compartment syndrome is a surgical emergency.

## Conclusions

Intravenous contrast extravasation, while uncommon, can lead to severe complications, including acute compartment syndrome. Awareness of this risk among all healthcare providers involved in imaging procedures is essential for early recognition and timely intervention. Prompt surgical decompression remains critical to preserving limb function and preventing irreversible soft tissue damage. Careful patient assessment, meticulous catheter placement, and close monitoring during contrast administration are important preventive measures. Ultimately, maintaining a high index of suspicion and ensuring a rapid multidisciplinary response are key to minimizing morbidity and achieving optimal patient outcomes.
